# Prevalence of multimorbidity in the Cypriot population; A cross-sectional study (2018–2019)

**DOI:** 10.1371/journal.pone.0239835

**Published:** 2020-10-26

**Authors:** Maria Kyprianidou, Demosthenes Panagiotakos, Antigoni Faka, Maria Kambanaros, Konstantinos C. Makris, Costas A. Christophi

**Affiliations:** 1 Cyprus International Institute for Environmental and Public Health, Cyprus University of Technology, Limassol, Cyprus; 2 Department of Nutrition and Dietetics, School of Health Sciences and Education, Harokopio University, Athens, Greece; 3 Department of Geography, School of Environment, Geography and Applied Economics, Harokopio University, Athens, Greece; 4 Department of Rehabilitation Sciences, Cyprus University of Technology, Limassol, Cyprus; Sciensano, BELGIUM

## Abstract

**Background:**

Multimorbidity is defined as the co-existence of two or more chronic conditions. As life expectancy is increasing so does the prevalence of multimorbidity. Our aim was to estimate the prevalence of multimorbidity in Cyprus and identify the most prevalent diseases.

**Methods:**

A representative sample of *n* = 1140 individuals over 18 years old was surveyed during 2018–2019. Demographic characteristics as well as the presence of chronic conditions, including mental disorders, were collected through a standardized questionnaire. Diseases were classified according to the International Classification of Diseases, 10^th^ Revision (ICD-10).

**Results:**

The age and gender standardized prevalence of multimorbidity was 28.6%. Multimorbidity was associated with age (p<0.001), with the highest rate observed among people aged 65+ years old (68.9%). Multimorbidity was higher in women than men (28.2% vs. 22.5%, p < .001) but similar in urban and rural regions (26.4% vs. 23.8%, p = 0.395). The most prevalent chronic diseases among people with multimorbidity were hyperlipidemia (44.7%), followed by hypertension (37.5%), gastric reflux (23.9%), and thyroid diseases (22.2%), while the most common combinations of diseases were in the circulatory and endocrine systems. The profile of the multimorbid individual indicated this to be a person at an older age with a higher BMI, a current smoker with a higher salary.

**Conclusions:**

More than one quarter of the general population of Cyprus has multimorbidity, and this rate is almost 70% among the elderly. Multimorbidity is relatively common even in younger ages too. This underlines the need for prevention strategies and health awareness programs for the entire population.

## Introduction

During the last decade, there has been an increasing interest in the clinical importance of multimorbidity, which is defined as the co-existence of two or more chronic conditions [1]. Multimorbidity is a relatively common condition which affects a considerable proportion of adults of all ages [1, 2], and as epidemiological studies have been noticed. It is even more common in the elderly, among whom the prevalence was estimated to be 60% in some populations [2, 3]. Of great interest is the fact that even in younger ages the prevalence of multimorbidity is relatively high and it has been reported that almost 50% of the people with multimorbidity are in fact younger than 65 years old [4]. Given that chronic illnesses are the leading cause of morbidity worldwide with several diseases being related to aging [3] and the fact that the World Health Organization (WHO) estimates that by 2030 life expectancy will be even higher than today, multimorbidity is emerging as a new global health challenge.

Multimorbidity is associated with reduced quality of life [3] and it can limit access to therapies and operative interventions [5] as well as increase disabilities and mortality [2, 6, 7]. Several factors have been identified that contribute to the multimorbidity burden, including socio-demographic factors, such as age [8–15], gender [16], education [17], and socio-economic status [4, 7, 18], body mass index (BMI) [7], quality of life [19], physical activity [7], smoking [20], as well as other psychological and environmental factors [3]. The onset of multimorbidity may occur 15–20 years earlier in individuals living in deprived areas as a result of poor quality of life to an increased risk for poor health outcomes, such as hospital re-admission, institutionalization, and mortality [21]. Similarly, several epidemiological studies have shown that multimorbid individuals have a poor quality of life along with disability, functional decline and high health care costs, and quality of life decreases with an increasing number of diseases [4]. Estimates of multimorbidity prevalence vary from 10% to over 90% from study to study [22, 23]. This wide variation is partly due to divergent dissimilar definitions, including the number of chronic conditions considered, the data sources and the data collection methods used (self-reported questionnaires, questionnaires with medical history obtained from a health professional, pharmacy databases to identify different conditions), and differences in demographic characteristics, in recruitment methods, and sample sizes. In addition, some studies focused only on specific chronic or mental health diseases or the presence of two or more chronic diseases from a smaller number of diseases [3].

According to the recent latest WHO data (2018), the life expectancy at birth in Cyprus, a high-income country in the Eastern Mediterranean, is 82.2 years (83.1 years for women and 78.4 years for men), which is among the highest in the EU, giving Cyprus a World Life Expectancy ranking of 28. Furthermore, the Cypriot population has several characteristics, which could be associated with the occurrence of a number of chronic diseases. For instance, Cyprus has a high prevalence of smoking (39% vs. 17%) (WHO, 2019) and overweight/obese people (60% vs. 38%) for men and women respectively (EUROSTAT, 2014).

Since assessment of prevalence of multimorbidity and its related factors in a population has important implications for prevention, diagnosis, treatment, and public health strategies, there is a need for generating relevant data. In addition, individuals with multiple chronic conditions have more health needs which should be recognized by the health system. The goal of this work was to assess the prevalence of multimorbidity in the adult population of Cyprus and create the profile of the multimorbid individual. Furthermore, this our study aimed to identify the most common diseases and the most frequent combinations of diseases in the Cypriot population.

## Methods

### Study design

This was a cross-sectional study.

### Setting

The referent population included all men and women over 18 years old, living in the five government-controlled municipalities of the Republic of Cyprus (Nicosia, Limassol, Larnaca, Paphos, and Ammochostos). Individuals living in nursing homes or those institutionalized were excluded. Data collection took place during May 2018-June 2019.

### Study size

The required sample size to estimate the prevalence of multimorbidity using a 95% confidence interval (CI) with a precision of 5%, assuming a true prevalence of 30% was *n* = 1,145.

### Sampling

A stratified sampling procedure, on a feasibility basis, was implemented to ensure that the sample matched the Cypriot population in three key demographic characteristics (age, sex, and region). Using the latest census data (2011) available, the referent population was divided into the five municipalities of Cyprus. Then, the population was further stratified according to the type of residence (urban, rural as provided by the National Bureau of Statistics), gender (male, female), and age group (18–24, 25–44, 45–64, 65+ years old). Recruitment occurred in public places and in houses throughout Cyprus; Nicosia (43% of the total Cypriot population), Limassol (27%), Larnaca (15%), Paphos (10%), and Ammochostos (5%). The study sample was similar to the general population with respect to region, age, and gender. The overall response rate was 90%.

### Bias

To account for potential biases during the enrollment of the participants, the sampling, although on a feasibility basis, was unrestricted by any conventional selection rules or procedures, and an effort was made to be as random as possible.

### Participants’ characteristics

The information was collected via a face-to-face interview by trained investigators, using a standardized questionnaire. Data included socio-demographic characteristics (i.e., age in years, gender, marital and educational status, and annual income), lifestyle habits (i.e., smoking, dietary habits, and physical activity), and a detailed medical history.

Marital status was recorded as never married, married/engaged, or separated/-divorced/widowed. Education level was classified into three categories commonly used in Cyprus and similar to a study on the Greek population [24], namely: (i) primary education (participants who completed only primary school—<7 years of schooling); (ii) secondary education (participants who completed middle or high school—7–12 years of schooling); (iii) higher education (participants who have a university degree—>12 years of schooling). Financial status was evaluated using the annual income and was classified as: (i) low (≤ €6,500); (ii) moderate (€6,500–19,500); and (iii) high (> €19,500). Current smokers included those who smoked at least one cigarette a day and not current smokers included never and former smokers. For the evaluation of physical activity, the International Physical Activity Questionnaire (IPAQ) was used as an index of weekly energy expenditure using frequency (times per week), duration (in minutes per time), and intensity of sports or other habits related to physical activity (in expended calories per time). Physical activity was defined as leisure-time activity of a certain intensity and duration, at least once/week during the past year, including “light” (expended calories < 4 Kcal/ min), “moderate” (expended calories 4–7 Kcal/ min), or “vigorous” (expended calories >7 Kcal/ min). Body mass index (BMI) was calculated as weight (in kilograms) divided by standing height (in meters squared); weight and height were measured using standard procedures. Obesity was then defined as BMI >29.9 kg/m^2^, overweight as BMI 25–29.9 kg/m^2^, normal as BMI 18.5–24.9 kg/m^2^, and underweight as BMI <18.5 kg/m^2^, according to the WHO classification.

The medical history of the participants included the presence, as diagnosed by a physician, of chronic conditions was performed via a face-to-face interview. The question used to obtain the medical history of the participants was: “Have you ever been diagnosed by a physician with any of the following chronic diseases? Choose all that apply”. Diseases were coded according to the International Classification of Diseases, 10^th^ Revision (ICD-10). The medical history considered 47 chronic diseases of all circulatory, digestive/excretory, endocrine, immune, nervous, renal/urinary, reproductive, respiratory, and skeletal/muscular systems, as well as neoplasms. Details are presented in **S1 Data**.

Multimorbidity was defined, according to a recent systematic review and meta-analysis that evaluated different definitions of multimorbidity, “*as any combination of chronic disease with at least one acute or chronic disease or biopsychosocial factor or somatic risk factor”* [19]. The no multimorbidity group included participants with no disease or only one disease reported.

### Ethics approval

The study was approved by the Cyprus National Bioethics Committee (CNBC) (ΕΕΒΚ ΕΠ 2018.01.123). The application, along with all relevant questionnaires, submitted to the CNBC outlined the study objectives and outcomes, the data collection process and data management, the use of the data, and the expected benefits. The researchers approached people at public places, including kiosks, supermarkets, malls, restaurants, village squares, public services, and universities. They also visited homes by knocking on people’s doors. There were always 2 trained researchers together. After explaining the purpose of the study, including the gap in the literature on the prevalence of multimorbidity in Cyprus and its association with potential risk factors, the researchers would point out that the study has been approved by the CNBC and that their participation would be anonymous as well as that they could stop participating at any time they wanted to. The researchers would then ask for the participants’ consent to participate (verbal) and then they would provide the questionnaire of the study for completion. All the details above were also included in the first page of the questionnaire.

### Statistical analysis

Continuous variables (e.g., age and BMI) are presented as mean ± standard deviation (SD) and categorical variables (e.g., age group, gender, geographical area, residency, marital status and educational level, salary, physical activity, smoking, and obesity group) as absolute and relative (%) frequencies. We assessed the prevalence of multimorbidity in the general population of Cyprus using age and gender standardized values by using the appropriate formula which evaluates the range in which the true value lies with a certain degree of probability, as well as the direction and strength of the demonstrated effect. The distributions of continuous variables were assessed for normality and the Student’s t-test was used for the comparison of baseline characteristics between the multimorbidity and no multimorbidity groups. The Pearson’s chi-squared test was applied to evaluate potential associations between categorical variables. The Cochran-Armitage test for trend was used to assess trends between categorical variables. Radar graphs were constructed to present the most commonly occurring combinations of chronic conditions. Discriminant classification analysis, with the calculation of Wilk’s lambda (the closer to 1, the better discriminating ability) and Fisher’s classification function coefficients was used to explore the patterns of characteristics of people with multimorbidity. Mixed effects multinomial regression was performed to evaluate the significance of different factors on level of multimorbidity after accounting for the age and sex distribution within the various sampling regions. All statistical hypotheses were two-sided with the statistical significance level set at α = 0.05. Statistical analysis was conducted using R studio version 1.2.1335 and STATA 14.0 statistical software (Stata Corp, College Station, TX, USA).

### Spatial analysis

Cyprus is characterized by several geographical disparities in the health care system distribution, in terms of the number of hospitals, health centres, physicians’ offices and practicing physicians. In order to assess the spatial distribution of multimorbidities in the general population in Cyprus, the parameter of spatial heterogeneity was accounted for and the analysis was based on the districts of Cyprus (Nicosia-the capital area- with a total population of 332,200; Limassol, 239,400; Larnaca, 144,900; Paphos, 91,300, and Ammochostos, 47,000). A mixed effects model was used to account for the age and sex distribution by region in order to evaluate the associations between multimorbidity prevalence and the healthcare-related environmental variables in the Cypriot districts. Geodatabase, spatial analyses and mapping were performed using the ArcGIS version 10.4 (ESRI Inc., Redlands, California, USA) and QGIS version 3.10.

## Results

### Participants’ characteristics

The mean age of the participants was 40 ± 16 years old for the *n* = 642 women and 42 ± 18 years old for the *n* = 497 men. Further details about the participants’ main characteristics are presented in **Table 1**.

**Table 1:** Characteristics of participants, overall and by multimorbidity group.

Characteristics	Overall (N = 1140)	Multimorbidity (N = 293)	No multimorbidity (N = 847)	*p*-value
Age (years)	40.8 ± 16.9	52.9 ± 18.0	36.6 ± 14.2	< .001
Age group				< .001
*18–24*	167 (14.7)	13 (7.8)	154 (92.2)	
*25–44*	524 (46.0)	84 (16.0)	440 (84.0)	
*45–64*	314 (27.6)	103 (32.8)	211 (67.2)	
*65+*	135 (11.8)	93 (68.9)	42 (31.1)	
Sex				< .001
*Male*	497 (43.7)	112 (22.5)	385 (77.5)	
*Female*	642 (56.4)	181 (28.2)	461 (71.8)	
Geographical area				0.179
*Nicosia*	493 (43.3)	131 (26.6)	362 (73.4)	
*Limassol*	311 (27.4)	89 (28.6)	222 (71.4)	
*Larnaka*	171 (15.0)	36 (21.0)	135 (79.0)	
*Paphos*	113 (9.9)	22 (19.5)	91 (80.5)	
*Ammochostos*	50 (4.4)	15 (30.0)	35 (70.0)	
Residency				0.395
*Urban*	864 (76.3)	228 (26.4)	636 (73.6)	
*Rural*	269 (23.7)	64 (23.8)	205 (76.2)	
Marital status				< .001
*Married*	616 (54.4)	194 (31.5)	422 (68.5)	
*Unmarried*	421 (37.2)	50 (11.9)	371 (88.1)	
*Divorced / Widowed*	96 (8.5)	46 (47.9)	50 (52.1)	
Educational status				< .001
*Primary education*	66 (5.8)	48 (72.7)	18 (27.3)	
*Secondary education*	338 (29.8)	87 (25.7)	251 (74.3)	
*Higher education*	729 (64.3)	157 (21.5)	572 (78.5)	
Salary (yearly average)				0.170
*Low* (≤ €6,500)	241 (21.3)	53 (22.0)	188 (78.0)	
*Middle* (€6,500– 19,500)	562 (49.7)	144 (25.6)	418 (74.4)	
*High* (> €19,500)	328 (29.0)	95 (29.0)	233 (71.0)	
Physically active	541 (47.8)	108 (20.00)	433 (80.0)	< .001
Current smoker	402 (35.5)	121 (30.1)	281 (70.0)	0.016
BMI (kg/m^2^)	25.0 ± 4.6	26.4 ± 4.9	24.5 ± 4.4	< .001
BMI group				< .001
*Underweight*	42 (3.7)	5 (11.9)	37 (88.1)	
*Normal*	565 (50.4)	112 (19.8)	453 (80.2)	
*Overweight*	362 (32.3)	105 (29.0)	257 (71.0)	
*Obese*	152 (13.6)	66 (43.4)	86 (56.6)	

### Distribution of multimorbidity

The overall age and gender standardized prevalence of multimorbidity was 28.6% (95% CI: 26.0, 31.2). The mean number of conditions was 1.07. The multimorbidity rate increased significantly with age (*p* for trend <0.001), was higher in women than in men (28% vs. 23%, p<0.001), in overweight/obese people (p<0.001), among divorced/widowed participants, in people who completed only primary school, among current smokers, and in people who were physically inactive (**Table 1**). No significant differences were observed between residents of urban and rural regions, among the five geographical areas of Cyprus, or among the salary categories.

**Fig 1:**
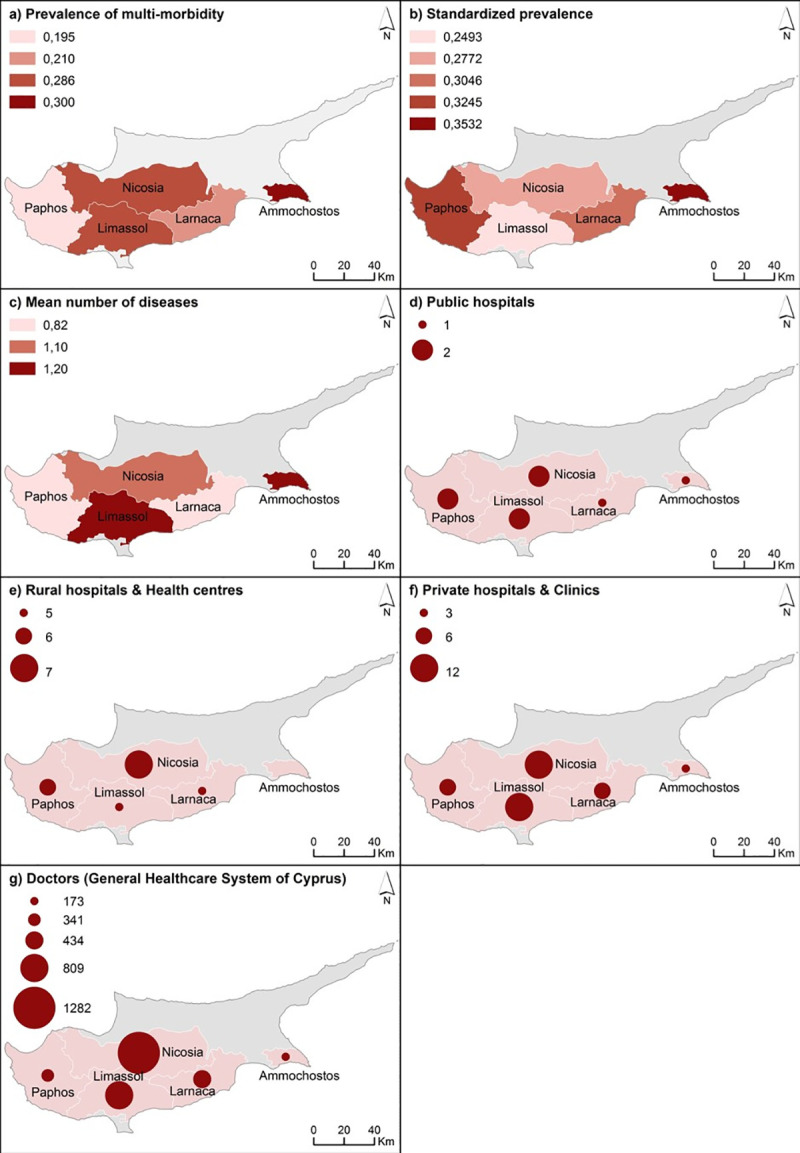
Spatial analysis of multi-morbidities by region of residence and level of urbanicity.

The prevalence and standardized prevalence of multimorbidity and the mean number of diseases are presented by region in **Fig 1** which also highlights the presence of general hospitals, health centers and private physicians’ office (as retrieved from the Cyprus Ministry of Health). The data suggests that the prevalence of multimorbidity was inversely correlated with the presence of health centers within a region (*p*<0.001), and positively correlated with the population density of practicing physicians (i.e., physicians per 10,000 population) (*p*<0.001).

### Decomposition of multimorbidity

**Fig 2:**
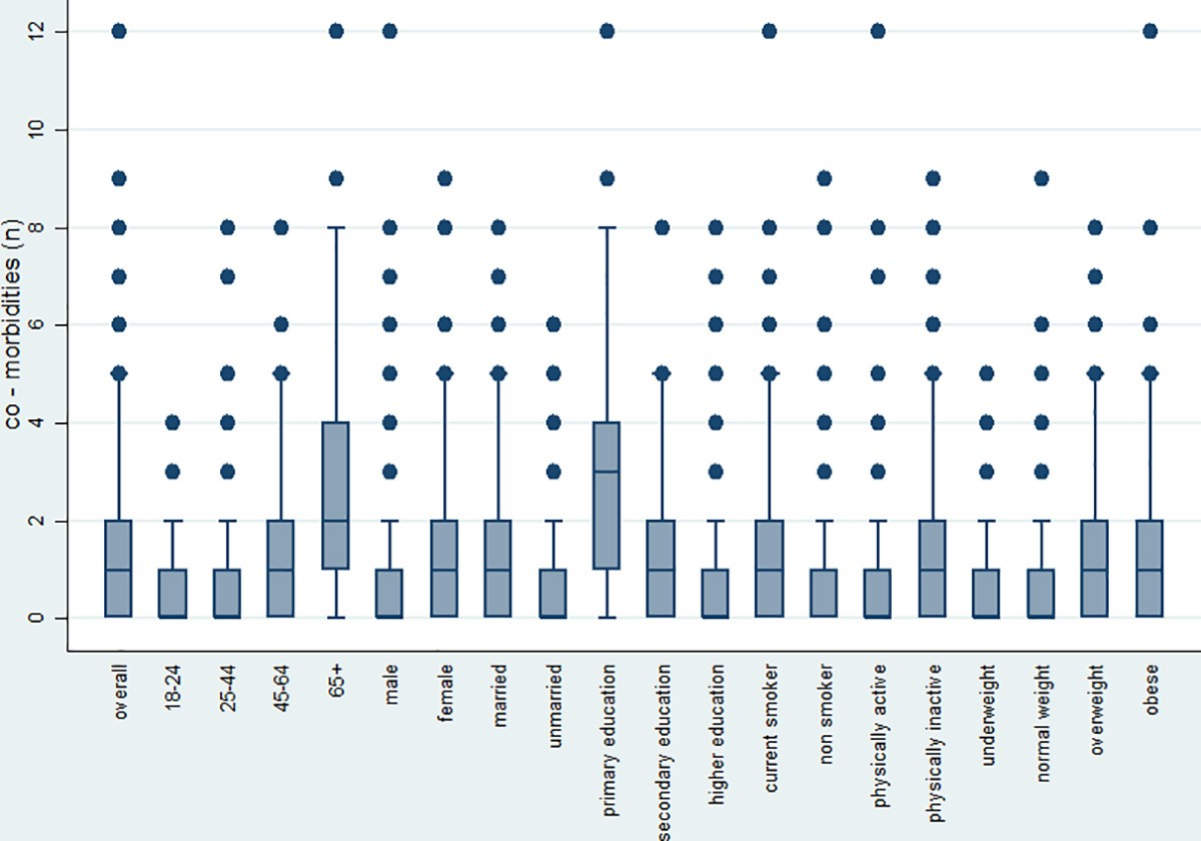
Distribution of co-morbidities overall and by age group, sex, marital and education status, smoking and physical activity level, and obesity status.

The median number of conditions per participant was 1 (quartiles, q_1_ = 0 and q_3_ = 2) with the maximum number being 12. **Fig 2** shows the median number of morbidities, by age group, sex, marital and education status, smoking and physical activity level, as well as obesity group. A higher median number of morbidities was observed in people who completed only primary education and in those aged 65+. We also identified that among the study participants 11.8% had 2 morbidities, 6.6% had 3 morbidities, 5.3% had 4 morbidities and only 0.03% had 5 morbidities.

The most prevalent chronic diseases were diseases of the circulatory system (with rates 25% and 61%, overall and among participants with multimorbidity, respectively), followed by the endocrine system (17% vs. 43%), and the digestive-excretory system (13% vs. 39%). Moreover, the crude prevalence of specific chronic diseases showed that the most prevalent condition was hyperlipidemia (17.4%), followed by hypertension (12.9%), thyroid diseases (8.5%), and gastric reflux (7.4%), with the figures increasing with age (*p*<0.001) (**Table 2**).

**Table 2:** Crude prevalence of chronic diseases by gender and age group.

Chronic diseases	Overall N = 1140	Men N = 497	Women N = 643
**Number of co-morbidities**	1.07 ± 1.49	0.98 ± 1.49	1.14 ± 1.48
**Hyperlipidemia** (E78.5)	198 (17.4)	98 (19.8)	100 (15.6)
*18–24*	5 (3.0)	0 (0.0)	5 (5.4)
*25–44*	44 (8.4)	26 (11.9)	18 (5.9)
*45–64*	87 (27.7)	37 (28.0)	50 (27.5)
*65+*	62 (45.9)	35 (48.0)	27 (44.3)
**Hypertension** (I10)	147(12.9)	83 (16.7)	64 (10.0)
*18–24*	0 (0.0)	0 (0.0)	0 (0.0)
*25–44*	15(2.9)	10 (4.6)	5 (1.6)
*45–64*	68 (21.7)	37 (28.0)	31 (17.0)
*65+*	64 (47.4)	36 (49.3)	28 (45.9)
**Thyroid diseases** (E02, E03.8, E03.9, E05.90, E07.9)	96 (8.4)	17 (3.4)	79 (12.3)
*18–24*	2 (1.2)	0 (0.0)	2 (2.2)
*25–44*	38 (7.3)	5 (2.3)	33 (10.8)
*45–64*	40 (12.7)	6 (4.6)	34 (18.7)
*65+*	16 (11.9)	6 (8.2)	10 (16.4)
**Gastric reflux** (K21)	84 (7.4)	34 (6.8)	50 (7.8)
*18–24*	3 (1.8)	1 (1.4)	2 (2.2)
*25–44*	33 (6.3)	12 (5.5)	21 (6.9)
*45–64*	25 (8.0)	12 (9.1)	13 (7.1)
*65+*	23 (17.0)	9 (12.3)	14 (23.0)
**Polycystic ovarian syndrome** (E28.2)	-	-	69 (6.1)
*18–24*	8 (4.8)	-	8 (8.6)
*25–44*	49 (9.3)	-	49 (16.0)
*45–64*	10 (3.2)	-	10 (5.5)
*65+*	2 (1.5)	-	2 (3.3)
**Asthma** (J45)	65 (5.7)	30 (6.0)	35 (5.5)
*18–24*	14 (8.4)	6 (8.1)	8 (8.7)
*25–44*	29 (5.5)	15 (6.9)	14 (4.6)
*45–64*	16 (5.1)	6 (4.6)	10 (5.5)
*65+*	6 (4.4)	3 (4.1)	3 (4.9)
**Irritable Bowel syndrome** (K58)	55 (4.8)	8 (1.6)	47 (7.3)
*18–24*	4 (2.4)	1 (1.4)	3 (3.2)
*25–44*	23 (4.4)	4 (1.8)	19 (6.2)
*45–64*	21 (6.7)	3 (2.3)	18 (9.9)
*65+*	7 (5.2)	0 (0.0)	7 (11.5)
**Depression** (F32, F33)	39 (3.4)	10 (2.0)	29 (4.5)
*18–24*	5 (3.0)	1 (1.4)	4 (4.3)
*25–44*	14 (2.7)	5 (2.3)	9 (2.9)
*45–64*	16 (5.1)	2 (1.5)	14 (7.7)
*65+*	4 (3.0)	2 (2.7)	2 (3.3)

**Fig 3:**
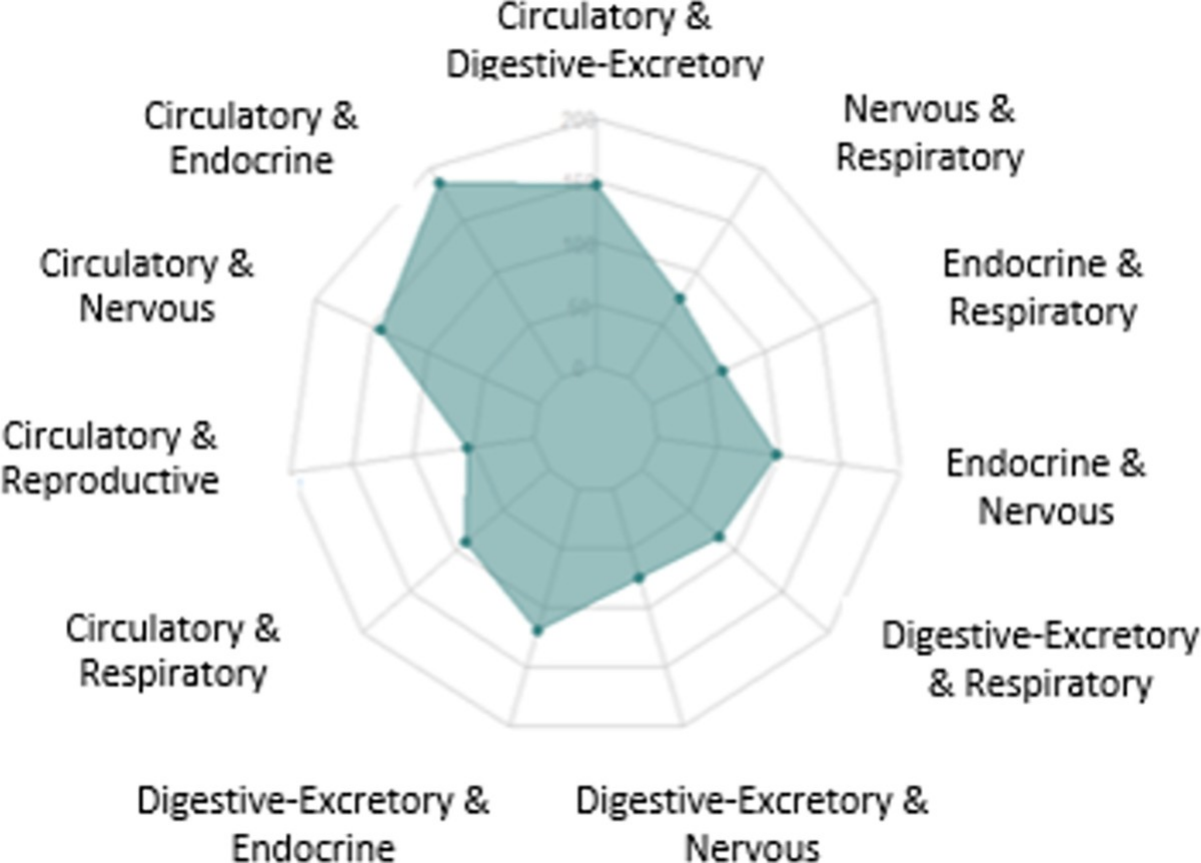
Combinations of human systems’ diseases among people with multimorbidity.

Among men, the most prevalent chronic diseases were hyperlipidemia, hypertension, and gastric reflux, while among women these were hyperlipidemia, thyroid diseases, and hypertension. The combinations of human systems’ diseases among people with multimorbidity are presented in **Fig 3**. The most prevalent conditions were diseases of circulatory/endocrine systems, followed by circulatory/digestive-excretory systems, and circulatory/nervous systems. Specifically, the most common combinations among people with multimorbidity were diseases of the circulatory and endocrine systems (25%), followed by circulatory and digestive-excretory systems (20%), circulatory and nervous systems (19%) (Fig 3). More specifically, the most prevalent combinations of chronic diseases among people with multimorbidity were hyperlipidemia-thyroid diseases (9%), hypertension- thyroid diseases (9%), and hyperlipidemia-gastric reflux (8%).

Hierarchical discriminant analysis, providing the Wilk’s *lambda* and standardized Fisher’s classification function coefficients revealed that the Wilk’s *lambda* was equal to 0.776 (*p*<0.001), a value close to 1, which indicates that the estimated model has good classification ability to multimorbidity classes. Discriminant analysis revealed that in individuals with 2-morbidities increased age, and higher BMI contributes more in their categorization as having 2-comorbidities compared to none or 1-morbidity. Similarly, in individuals with 3-morbidities increased age, current smoking and higher salary are the dominant factors to characterize those participants, whereas, among individuals with >3-morbidities, male sex, higher salary and increased BMI seems to characterize them better.

The coefficients of variation within each region were 75%, 73%, 72%, 63%, and 73% for Nicosia, Limassol, Larnaka, Paphos, and Ammochostos, respectively. Moreover, the variability of between regions was only 1%. Furthermore, mixed effects multinomial regression showed that the presence of 2 morbidities in an individual compared to none or 1 morbidity was associated with increased age and BMI. Similarly, the presence of 3 morbidities was associated with being a female, a smoker, as well as with increased age and BMI. Finally, having 3 or more morbidities was associated with increased age, being female, unmarried and completed a secondary education (**Table 3**).

**Table 3:** Mixed effects multinomial regression of multimorbidity level after accounting for all other variable effects.

Characteristics	Multimorbidity level (base outcome = 0 or 1 morbidities)		
	2	3	>3
Age (per 1 year)	0.04 (0.03, 0.06)*	0.06 (0.04, 0.08)*	0.08 (0.06, 0.10)*
Men	-0.04 (-0.86, 0.04)	-1.24 (-1.90, -0.57)*	-1.14 (-1.73, -0.55)*
Marital status			
*Unmarried*	0.13 (-0.46, 0.72)	-0.67 (-1.66, 0.31)	0.98 (0.22, 1.75)
*Divorced/widowed*	0.15 (-0.50, 0.81)	-0.31 (-1.19, 0.58)	0.57 (-0.11, 1.26)
Educational status			
*Secondary*	-0.27 (-1.18, 0.64)	-0.18 (-1.31, 0.95)	-1.23 (-2.12, -0.35)
*Higher*	-0.24 (-1.22, 0.73)	0.17 (-1.05, 1.39)	-0.81 (-1.78, 0.16)
Salary			
*Middle*	0.54 (-0.06, 1.15)	0.24 (-0.56, 1.05)	0.41 (-0.33, 1.14)
*High*	0.31 (-0.41, 1.02)	0.29 (-0.62, 1.19)	0.73 (-0.12, 1.57)
Physically active	-0.13 (-0.55, 0.28)	-0.05 (-0.62, 0.51)	-0.39 (-0.92, 0.14)
Current smoker	0.20 (-0.22, 0.62)	0.59 (0.02, 1.16)	0.23 (-.30, 0.76)
BMI (per 1 kg/m^2^)	0.05 (0.01, 0.09)*	0.07 (0.01, 0.12)*	0.04 (-0.02, 0.09)

*p<0.05.

## Discussion

To the best of our knowledge, this is the first large-scale analysis investigating multimorbidity in Cyprus using a representative sample of the general adult population and considering 47 different chronic conditions. The overall age and gender standardized prevalence of multimorbidity was 28.6%. This is comparable to studies with a cross-sectional design and similar definition of multimorbidity carried out in Scotland [4], Australia [23], Serbia [25], and Brazil [10]. The prevalence in Cyprus seems to be lower than Canada [26], Switzerland [27], and Indonesia [28], but higher than South Asia [29], China [16], and Iran [30]. About half of the study participants did not report suffering from any of the chronic diseases considered, something that is similar to results reported for Finland [29] and Italy [31].

The fact that the prevalence of multimorbidity increases with age is consistent with observations from other studies around the world [16, 23, 32]. Given the aging population of Cyprus and the fact that multimorbidity increases with age, the prevalence of multimorbidity is expected to rise even higher in the future. Moreover, multimorbidity was inversely correlated with the number of health centers in each region. This finding should motivate the public health authorities of Cyprus in setting priorities in the organization of primary and secondary health care, especially in light of the new general health system currently being implemented. Multimorbidity was also positively correlated with the number of physicians per region, which is encouraging as each patient may be receiving the relevant treatment.

Our study suggests that multimorbidity is common not only among the elderly but also among people of younger ages. Other cross-sectional studies which investigated the prevalence of multimorbidity using medical records or self-reported questionnaires also reported this finding [33]. We further observed that the prevalence of multimorbidity was higher in women than in men which has also been described in other studies [16, 34]. Given the relatively high prevalence of endocrine disorders, and that in our population a higher percentage of women than men were physically inactive, and had completed primary school only, which was also associated with the presence of multimorbidity, may in part explain the higher prevalence of multimorbidity in women.

We found bivariate associations between socio-demographic characteristics, lifestyle factors, such as marital status, educational level, exercise, smoking, and BMI, and the prevalence of multimorbidity but there was no association with geographical area, residency, and salary. The higher prevalence of multimorbidity in people with lower education concurs with previous results published elsewhere [16, 17]. Moreover, discriminant analysis suggested that in individuals with 2-morbidities increased age, and higher BMI are more dominant factors compared to individuals with none or 1-morbidity category. Furthermore, in individuals with 3-morbidities increased age, current smoking and higher salary are the dominant factors to characterize those participants while among individuals with >3-morbidities, male sex, higher salary and increased BMI seems to better characterize them. The higher salary as a dominant factor was an unexpected finding, but was also reported in another epidemiological study in China [16]. We defined multimorbidity as “two or more” morbidities which is the definition most commonly used, however, especially when highly prevalent conditions (e.g., hypertension, hyperlipidemia) are present in the population, using a higher cut-off to define multimorbidity may be preferred. Hence, we estimated the prevalence of study participants having 2, 3, 4, 5 and more than 5 morbidities specifically something that showed that the largest number of multimorbidity individuals has 2 morbidities while the cumulative frequency no longer increases after the cut-off of 5 morbidities.

The main chronic diseases, as reported in the literature, are cardiovascular diseases, chronic kidney disease, osteoarthritis, endocrine and metabolic diseases, and mental disorders. Our results are in agreement with this, suggesting that the majority of chronic diseases involve the cardiovascular and endocrine system. We identified that hyperlipidemia is prevalent in our study population, similar to studies in China, Brazil, Switzerland, and Indonesia [10, 18, 28, 35], though those studies used dyslipidemia and hypercholesterolemia as their definitions. Hypertension is also quite prevalent in the Cypriot population which is consistent with reports from other cross-sectional studies [28, 36–38].

We reported that the most prevalent combinations of chronic diseases were hyperlipidemia/ thyroid diseases and hyperlipidemia/ gastric reflux. On the one hand, thyroid dysfunction can have an important effect on lipid profile [39] and it has been known that the thyroid hormones have an effect on cholesterol synthesis and metabolism and, more specifically, hypothyroidism has been associated with increased levels of triglycerides and cholesterol [40]. On the other hand, a diet high in fat and calories, which is associated with obesity, usually co-exists with gastroesophageal reflux disease and with high cholesterol levels. However, there is no scientific evidence of a direct cause and effect relationship between gastroesophageal reflux disease and hyperlipidemia, so further research is needed to investigate this relationship.

Our study has some limitations. First, the cross-sectional design used means that only associations between the groups of interest could be examined and not causal relationships. Furthermore, the severity of the disease is not considered, and all chronic diseases included in the study had equal weight in the calculation of multimorbidity and the face-to-face interviews in the medical history assessment could imply social desirability bias. Survey weighting was not applied and institutionalized individuals were excluded from sampling, although, a high prevalence of chronic diseases and multimorbidity has been identified in this group of people. This may have underestimated the prevalence of multimorbidity in the total population but strengthens our results among free living people. At the same time, the study has several strengths as this is a large population-based study using a representative sample of both men and women from all ages (18+) and geographical areas of Cyprus. Other strengths include the collection of detailed data on the participants as well as information on a multitude of diseases.

In conclusion, multimorbidity is relatively common in the adult Cypriot population, not only among the elderly but also in younger ages, which underlines the need for prevention strategies and relevant programs for the entire population. The appropriate management of long-term disorders is a key challenge for health systems, and it is important to focus on the associations beyond chance or patterns of diseases. Knowledge about multimorbidity has important implications for prevention, diagnosis, treatment, and prognosis strategies, hence, future programs and practice guidelines in Cyprus and elsewhere should take into account the common patterns of multimorbidity.

## Supporting information

S1 Data (XLSX)
